# Tailoring the Topological Charge of a Superposition of Identical Parallel Laguerre–Gaussian Beams

**DOI:** 10.3390/mi13122227

**Published:** 2022-12-15

**Authors:** Victor V. Kotlyar, Alexey A. Kovalev, Elena S. Kozlova, Alexandra A. Savelyeva

**Affiliations:** 1 Image Processing Systems Institute of the RAS, Branch of FSRC “Crystallography & Photonics” of the RAS, 151 Molodogvardeyskaya St., Samara 443001, Russia; 2 Technical Cybernetics Department, Samara National Research University, Moskovskoye Shosse 34, Samara 443086, Russia

**Keywords:** optical computing machine, topological charge, Laguerre–Gaussian beam, superposition of parallel beams

## Abstract

In optical computing machines, data can be transmitted by optical vortices, and the information can be encoded by their topological charges. Thus, some optical mechanisms are needed for performing simple arithmetic operations with the topological charges. Here, a superposition of several parallel identical Laguerre–Gaussian beams with single rings is studied. It is analytically and numerically shown that if the weighting coefficients of the superposition are real, then the total topological charge of the superposition is equal to the topological charge of each component in the initial plane and in the far field. We prove that the total topological charge of the superposition can be changed by the phase delay between the beams. In the numerical simulation, we demonstrate the incrementing and decrementing the topological charge. Potential application areas are in optical computing machines and optical data transmission.

## 1. Introduction

Optical vortices constitute a great family of light fields, which is actively studied over 30 years [1]. The studies include various aspects, including direct generation in lasers [2], interaction with matter [3], and propagation and focusing [4]. Light fields with optical vortices are usually characterized by the orbital angular momentum (OAM) [5] and the topological charge (*TC*) [6]. In a number of studies, the topological charge (*TC*) of a superposition of parallel optical vortices (OVs), and in particular, parallel Laguerre–Gaussian (LG) beams, was studied. This problem has been of interest since 2000, when the number and location of OVs in a superposition of two parallel Gaussian beams with embedded OVs were studied in [7]. In [7], a transcendental equation was obtained analytically for determining the position of OVs. However, it is applicable only for cases when the vortices in both beams are of the first order. It was also shown that when two beams are separated by a certain critical distance, negative-order vortices appear along with positive-order vortices. Later, in [8], using the analysis of forks in the interference pattern of two parallel LG beams, it was shown that varying the distance between the beams changes the arrangement of screw dislocations in the superposition. In addition, the same authors [9] showed that the number of vortices in the superposition of two parallel LG beams can change during propagation in space, although the total *TC* remains unchanged. In [10], the superposition of two off-axis optical vortices, but with orthogonal polarization, was studied. Instead of the dynamics of phase singularities, this paper studied the dynamics of polarization singularities and the position of C-points as a function of the distance between vortices, their *TC*, and the phase delay between them. In [11], the interference of two off-axis Gaussian beams with different curvatures of the wave front was also considered. The conditions for vortex dipoles (two OVs of opposite orders) formation were obtained. In [12], the coherent and the incoherent superposition of two parallel partially coherent OVs were studied. It was shown that the type of superposition, the distance between the beams, the propagation distance, and the coherence parameter affect the number and location of coherence vortices. The number of vortices, however, is determined only numerically. Study [13] considered OVs that are formed in a superposition of off-axis vortices during a nonlinear process of three-wave mixing. The number of vortices and their *TC*s were established in some particular cases. In a recent article [14], the interaction of parallel Bessel–Gaussian beams was considered. The dependence of formation, annihilation, and splitting of OVs on the displacement of the beams from the optical axis, on their *TC*, and on the phase difference between them was studied. It was shown that the total *TC* of such a composite field is not necessarily equal to the sum of the *TC*s of the composite beams. In [15], it was shown how to calculate the *TC* of a superposition of only two parallel LG beams. In particular, in [15], it was analytically shown that if two beams have the same *TC*, for example, *m*, then the superposition of such beams with arbitrary distance between them will also have a *TC* equal to *m*.

In this paper, we generalize the results on a superposition of a finite number of parallel one-ring identical LG beams. Moreover, we will show that if the weight coefficients of such a superposition are real (that is, all LG beams have the same phase, but can have different amplitudes), then the superposition *TC* is equal to the *TC* of each beam, that is, *m*. It has already been proven earlier that the power-normalized OAM of such a superposition is also equal to the OAM of one LG beam in the superposition, that is, also *m* [16].

## 2. *TC* of the Superposition of Identical Parallel LG Beams in the Initial Plane

We consider a superposition of *N* parallel identical one-ring LG beams in the initial plane:

(1) $$E_{m}\left(x,y\right)=\sum_{n=1}^{N}c_{n}{\left(re^{i\varphi }-r_{n}e^{i\varphi_{n}}\right)}^{m}\exp\left(-r^{2}-r_{n}^{2}+2rr_{n}\cos\left(\varphi -\varphi_{n}\right)\right),$$

where (*x*, *y*) and (*r*, *φ*) are the Cartesian and the polar coordinates (*x* = *r* cos *φ*, *y* = *r* sin *φ*), respectively.

The *TC* of each beam in (1) is equal to *m*, the waist radius is included in the radial variable *r*: *r*/*w*. We assume that the weight coefficients $c_{n}$ in (1) are real numbers. Polar coordinates of beam centers are $\left(r_{n},\;\varphi_{n}\right)$. The *TC* of superposition (1) will be calculated using the Berry formula [6]:

(2) TC=12πlimr→∞Im∫02πdφ∂E(r,φ)/∂φE(r,φ).

where lim means the limit at r→∞, and Im is the imaginary part of a complex number.

Substituting (1) into (2), we obtain:

(3) TC=12πlimr→∞Im∫02π[∑n=1Ncn(reiφ−rneiφn)me−rn2+2rrncos(φ−φn)×(imreiφreiφ−rneiφn−2rrnsin(φ−φn))][∑n=1Ncn(reiφ−rneiφn)me−rn2+2rrncos(φ−φn)]−1dφ=m−2r2πIm∫02π∑n=1Ncnrnsin(φ−φn)(reiφ)me−rn2+2rrncos(φ−φn)∑n=1Ncn(reiφ)me−rn2+2rrncos(φ−φn)dφ=m−2r2πIm∫02π∑n=1Ncnrnsin(φ−φn)e−rn2+2rrncos(φ−φn)∑n=1Ncne−rn2+2rrncos(φ−φn)dφ=m.

In (3), the imaginary part of the last integral is equal to zero, since it is real. It follows from (3) that the *TC* of the superposition of parallel identical single-ring LG beams with numbers (0, *m*) is equal to *m* in the initial plane.

## 3. *TC* of the Superposition of Parallel Identical LG Beams with Different Weight Coefficients in the Far Field

We propose that there is a superposition of *N* identical one-ring LG beams displaced from the optical axis in the initial plane. Then, the complex amplitude in the initial plane is equal to:

(4) $$E_{m}\left(x,y\right)=\sum_{n=1}^{N}c_{n}{\left\{\frac{\sqrt{2}}{w_{0}}\left[\left(x-a_{n}\right)+i\left(y-b_{n}\right)\right]\right\}}^{m}\exp\left[-\frac{{\left(x-a_{n}\right)}^{2}+{\left(y-b_{n}\right)}^{2}}{w_{0}^{2}}\right],$$

where (*x*, *y*) are Cartesian coordinates in the initial plane, *w*_0_ is Gaussian beam waist, (*a_n_*, *b_n_*) are coordinates of beam centers, and *c_n_* are superposition coefficients. In contrast to (1), in (4), the superposition beams are taken with complex weight coefficients *c_m_* and the Gaussian beam waist radius is explicitly distinguished.

In the far zone, the displacement of each LG beam turns out to be a slope of the wavefront, that is, in the far zone, the LG beams become axial, but with slopes. Therefore, the complex amplitude of the entire superposition in the far zone has the form:

(5) $$E_{m}\left(r,\varphi ,z>>z_{0}\right)=\exp\left(-\frac{r^{2}}{w_{0}^{2}}\right){\left(\frac{\sqrt{2}}{w_{0}}re^{i\varphi }\right)}^{m}\sum_{n=1}^{N}c_{n}\exp\left(ika_{n}r\cos\varphi +ikb_{n}r\sin\varphi \right).$$

where (*r*, *φ*) are far-field polar coordinates. According to the formula of M.V. Berry [6], *TC* is equal to

(6) TC=12πlimr→∞Im∫02π∑n=1Ncn∂∂φ[exp(ikanrcosφ+ikbnrsinφ)]∑n=1Ncnexp(ikanrcosφ+ikbnrsinφ)dφ    +12πlimr→∞Im∫02π∂∂φ[(reiφ)m](reiφ)mdφ.

Reducing the common factors in the numerator and denominator, we obtain further:

(7) TC=m+12πlimr→∞Im∫02π∑n=1Ncn∂∂φ[exp(ikanrcosφ+ikbnrsinφ)]×[∑n=1Ncnexp(ikanrcosφ+ikbnrsinφ)]−1dφ.

The second term in (7) is the *TC* of some additional field of the form (without a Gaussian envelope).

(8) $$E_{\mathrm{add}}\left(x,y\right)=\sum_{n=1}^{N}c_{n}\exp\left(ika_{n}x+ikb_{n}y\right).$$

Since the numbers *a_n_*, *b_n_*, *c_n_* are arbitrary, Formula (8) can describe a wide class of light fields. In particular, the additional field can be a vortex and therefore give an additional *TC*. For example, if we take *N* = 4, *c*_1_ = −*i*, *c*_2_ = 1, *c*_3_ = *i*, *c*_4_ = −1, *a*_1_ = −*a*_3_ = *a*, *a*_2_ = *a*_4_ = 0, *b*_1_ = *b*_3_ = 0, *b*_2_ = −*b*_4_ = *a*, we then obtain an additional field in the form

(9) $$E_{\mathrm{add}}\left(x,y\right)=2\sin\left(kax\right)+2i\sin\left(kay\right),$$

which is near the center, approximately

(10) $$E_{\mathrm{add}}\left(x\approx 0,y\approx 0\right)=2ka\left(x+iy\right),$$

that is, it contains a vortex of the first order. If all coefficients *c_n_* are real in superposition (4), then we can show that

(11) $$E_{\mathrm{add}}^{*}\left(x,y\right)=E_{\mathrm{add}}(-x,-y).$$

From (11), it follows that if there is a zero amplitude at some point (*x*_null_, *y*_null_), then the amplitude is also zero at the point (−*x*_null_, −*y*_null_), and near zero, the amplitude is complex conjugate. That is, there is a “conjugate” vortex for each vortex in the field (8). The *TC*s of these “conjugate” vortices compensate each other, and therefore the *TC* of the field (8) with real coefficients *c_m_* is equal to zero. Expression (11) is proved simply:

(12) $$E^{*}\left(u,v\right)=\sum_{n=1}^{N}c_{n}\exp\left(-ixu-iyv\right)=\sum_{n=1}^{N}c_{n}\exp\left(ix\left(-u\right)+iy\left(-v\right)\right)=E\left(-u,-v\right).$$

There is also a physical reason why the field (8) cannot have other *TC* than zero. Indeed, the amplitude of the form (8) is formed in the Fourier plane (in the focus of a spherical lens) by a light field, which in the initial plane consists of *N* point sources with different amplitudes, but the same phase. A light field whose amplitude is a real function can only create OVs in pairs with +*p* and −*p TC*s. This also follows from the fact that an OV that has passed through the amplitude mask does not change its *TC* [17,18].

If the *TC* of the superposition (1) in the initial plane and in the far zone is the same and equals *m*, then in any other plane it is equal to *m*, if the coefficients *c_n_* are real.

## 4. Modeling

**Figure 1:**
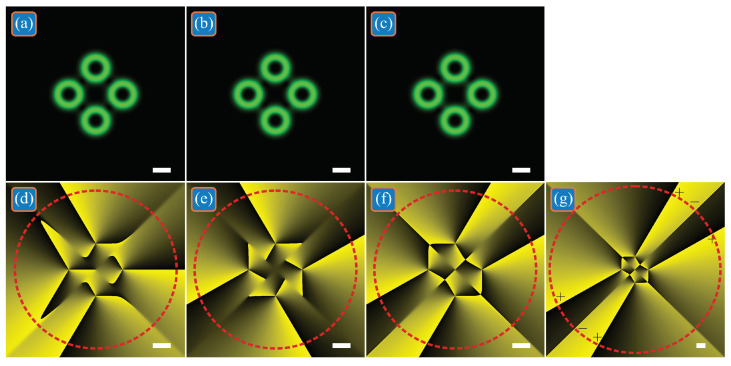
Intensities and phases of three superpositions of off-axis single-ring LG beams, in which the total *TC* is the same as that of each beam (**a**,**d**), or differs from the *TC* of each beam by +1 (**b**,**e**) and by −1 (**c**,**f**,**g**) due to the presence of complex weight coefficients in the superposition. Black and green intensity means zero and maximal values, respectively. Black and yellow phase means 0 and 2π, respectively. Here and in all other figures, red dashed circle on the phase distributions denotes the circle of the *TC* computation, whereas white scale marks in the right bottom denote 1 mm.

For example, Figure 1 shows the intensities and phases of three superpositions of off-axis single-ring LG beams with the following parameters: the wavelength is λ = 532 nm; the waist radius of all beams is *w*_0_ = 0.5 mm; the number of LG beams is *N* = 4; the *TC* of each of them is *m* = 3; the centers of these beams (in Cartesian coordinates) are (*a*_1_, *b*_1_) = (*r*_0_, 0), (*a*_2_, *b*_2_) = (0, *r*_0_), (*a*_3_, *b*_3_) = (–*r*_0_, 0), (*a*_4_, *b*_4_) = (0, −*r*_0_), where *r*_0_ = 3*w*_0_; the superposition coefficients for LG beams are *c*_1_ = *c*_2_ = *c*_3_ = *c*_4_ = 1 (Figure 1a,d), *c*_1_ = −*i*, *c*_2_ = 1, *c*_3_ = *i*, *c*_4_ = −1 (Figure 1b,e), and *c*_1_ = −*i*, *c*_2_ = −1, *c*_3_ = *i*, *c*_4_ = 1 (Figure 1c,f,g); the computational domain is restricted by |*x*|, |*y*| ≤ *R*, where *R* = 5 mm (Figure 1a–f) and *R* = 10 mm (Figure 1g); the radius of the circle for calculating the *TC* is *R*_1_ = 4.5 mm (Figure 1d–f) and *R*_1_ = 9.5 mm (Figure 1g); the grid size in each direction is *N* = 1024. When all superposition coefficients are the same (Figure 1a,d), the phase distribution becomes asymmetric, but the total *TC* of four LG beams turns out to be the same as for each of them: *TC* = 3.0042 ≈ 3. If we choose the coefficients, as in Figure 1b,e, then despite their dissimilarity, the phase distribution is symmetrical around the center and the total *TC* changes and turns out to be equal to *TC* = 4.0003 ≈ 4. For the other coefficients (Figure 1c,f,g), the total *TC* also changes and is equal to *TC* = 5.9234 ≈ 6 (Figure 1f). However, according to Equation (2), the *TC* is computed over an infinite-radius circle, and thus accounts all the vortices in the light field (or, equivalently, the 2π phase jumps in the beam periphery). Computation indicates that the figure size 2*R* = 10 mm is sufficient to account for all phase jumps in Figure 1d,e, but insufficient for Figure 1f. Figure 1g illustrates the phase distribution in a wider area (2*R* = 20 mm). In the periphery, there are four phase jumps by 2π and two phase jumps by −2π (denoted by ‘+’ and by ‘–’, respectively). Thus, the *TC* should be equal to (4 × 2π − 2 × 2π)/2π = 2. Numerical computation confirms it and yields the value *TC* = 1.9975 ≈ 2, which, in contrast with Figure 1b,e, is less than the *TC* of the constituent LG beams. Thus, the superposition coefficients *c*_1_ = −*i*, *c*_2_ = 1, *c*_3_ = *i*, *c*_4_ = −1 (Figure 1b,e) increment the *TC* of the whole superposition, whereas the coefficients *c*_1_ = −*i*, *c*_2_ = −1, *c*_3_ = *i*, *c*_4_ = 1 (Figure 1c,f,g) decrement this *TC*.

**Figure 2:**
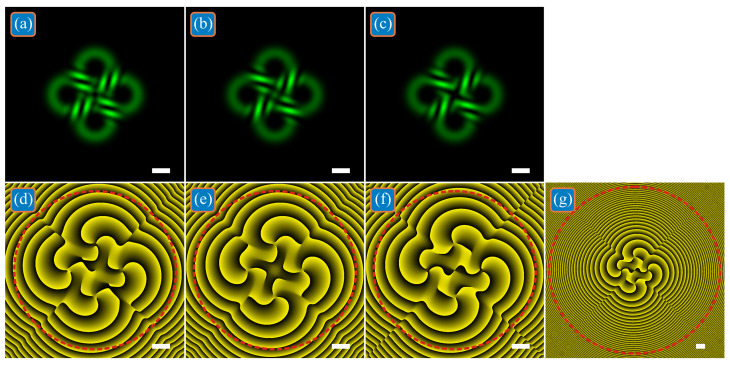
Intensities (**a**–**c**) and phases (**d**–**g**) of three superpositions of off-axis single-ring LG beams with the initial distribution shown in Figure 1 at the Rayleigh distance. The total *TC* of the superpositions is the same as that of each constituent beam (**a**,**d**), or differs from the *TC* of each beam by +1 (**b**,**e**) and by −1 (**c**,**f**,**g**) due to the presence of complex weight coefficients in the superposition.

When propagating in free space, four LG beams expand and begin to interfere with each other. Figure 2 shows the intensities and phases of the beams from Figure 1 with the same parameters, but at the Rayleigh distance *z* = *z*_0_ = *kw*_0_^2^/2 ≈ 1.476 m. When all superposition coefficients are equal to each other (Figure 2a,d), the total *TC* of four LG beams remains equal to three: *TC* = 2.9968 ≈ 3. For the beam in Figure 2b,e, the total *TC* remains equal to four: *TC* = 3.9903 ≈ 4. For the beam in Figure 2c,f, the total *TC* is equal to six (*TC* = 5.9148 ≈ 6), but, again, choosing a wider domain (Figure 2g) yields the value *TC* = 1.9713 ≈ 2.

**Figure 3:**
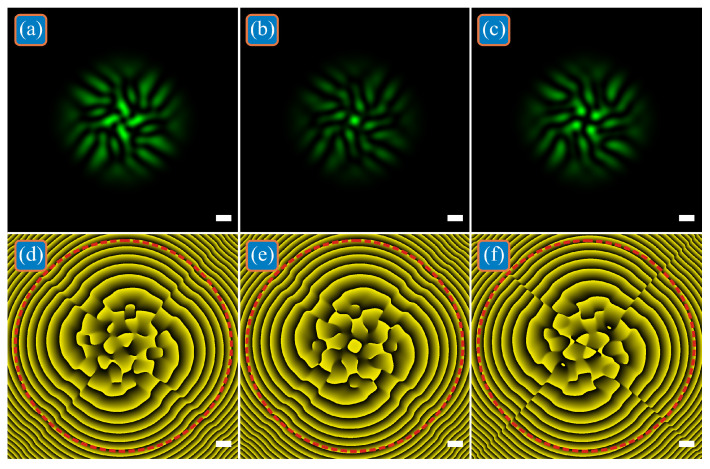
Intensities (**a**–**c**) and phases (**d**–**f**) of three superpositions of off-axis single-ring LG beams with the initial distribution shown in Figure 1 at triple Rayleigh distance (far zone). The total *TC* of the superpositions is the same as that of each constituent beam (**a**,**d**), or differs from the *TC* of each beam by +1 (**b**,**e**) and by −1 (**c**,**f**) due to the presence of complex weight coefficients in the superposition.

In the far zone, all four LG beams mix with each other, and the distributions of their intensity and phase are shown in Figure 3. All calculation parameters are the same as in Figure 1, but the propagation distance *z* = 3*z*_0_ ≈ 4.429 m, computational area is limited by |*x*|, |*y*| ≤ *R*, where *R* = 7.5 mm, circle radius for calculating *TC* is *R*_1_ = 7 mm. When all superposition coefficients are the same (Figure 3a,d), the total *TC* of four LG beams remains equal to three: *TC* = 2.9875 ≈ 3. For the beam in Figure 3b,e, the total *TC* is incremented and is equal to four: *TC* = 3.9760 ≈ 4. For the beam in Figure 3c,f, the total *TC* is decremented and is equal to two: *TC* = 2.0036 ≈ 2.

**Figure 4:**
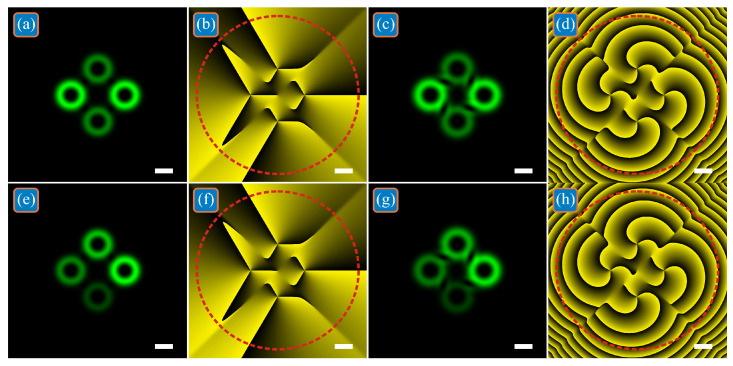
Intensities (**a**,**c**,**e**,**g**) and phases (**b**,**d**,**f**,**h**) of two superpositions of off-axis single-ring LG beams with different powers in the initial plane (**a**,**b**,**e**,**f**) and at the half of the Rayleigh distance (**c**,**d**,**g**,**h**). The superposition coefficients are *c*_1_ = *c*_3_ = 1, *c*_2_ = *c*_4_ = 1/2^1/2^ (**a**–**d**) and *c*_1_ = 1, *c*_2_ = 3^1/2^/2, *c*_3_ = 2^1/2^/2, *c*_4_ = 1/2 (**e**–**h**).

It should be noted that all LG beams in the superpositions shown in Figure 1, Figure 2 and Figure 3 have the same power. However, it follows from the theory above that if all superposition coefficients are real then the *TC* does not change even in the case of a superposition of LG beams with different power (LG beams are added in phase or in antiphase). Therefore, Figure 4 shows two such superpositions. In one of them, the LG beams on the horizontal axis are twice as powerful as the LG beams on the vertical axis: *c*_1_ = *c*_3_ = 1, *c*_2_ = *c*_4_ = 1/2^1/2^ (Figure 4a–d). In another superposition, the beam power decreases in a circle: *c*_1_ = 1, *c*_2_ = 3^1/2^/2, *c*_3_ = 2^1/2^/2, *c*_4_ = 1/2 (Figure 4e–h). Other calculation parameters are the same as in Figure 1, but the propagation distance *z* = 0 (Figure 4a,b,e,f) and *z* = *z*_0_/2 (Figure 4c,d,g,h). The numerically calculated *TC* for both beams at both distances along the optical axis turned out to be equal to three: *TC* = 3.0037 (Figure 4a,b), *TC* = 2.9995 (Figure 4c,d), *TC* = 3.0035 (Figure 4e,f), *TC* = 2.9993 (Figure 4g,h).

## 5. Discussion

In this section, we briefly consider the applications of the obtained results as well as the implementation issues. The potential application areas are optical data transmission and performing simple arithmetic operations in optical computing machines where data are carried by vortex light beams and are encoded by the topological charges [19,20].

In wireless optical data transmission, the results can be used by generating considered superpositions of LG beams using a spatial light modulator (SLM) and by identifying the incoming signals by the superposition’s topological charge using a Shack–Hartmann wavefront sensor. According to Figure 3, simply adding phase delays between the LG beams allows the *TC* of the whole superposition to change, whereas using the superposition of the LG beams instead of separate LG beams increases the resistance to the turbulence-induced distortions, adds degrees-of-freedoms in data encoding, and improves the data security, since the *TC* of a single LG mode can be determined simply by the radius of the light ring, while the *TC* of the superposition depends not only on the radii of the constituent beams, but also on the phase delays between them.

In optical computing, for designing compact devices, the LG beams should be localized in guiding microstructures rather than propagating in free space. However, the implementation of LG beam propagation in on-chip devices is now challenging for typical semiconductor-manufacturing processes, since most of integrated optical waveguides do not have circular symmetry in their transverse geometries in contrast to optical fibers. However, such techniques are developed now. For instance, a ±1st-order vortex can be implemented in a rectangular-shaped waveguide as a superposition of the TE01 and TE10 modes with a phase delay of π/2 [21]. In [22], an integrated cross-shaped waveguide structure to support high-order OAM modes up to the fourth order was proposed. This waveguide was designed for a wavelength of 1550 nm and has transverse sizes of just 1.626 μm × 1.504 μm. The obtained results show that simple free-space mixing of the LG beams, coming out from such structures, can generate light fields with incremented or decremented *TC*, although we did not investigate the influence of the mode purity of the LG beams.

## 6. Conclusions

Thus, we have proven that the superposition of identical single-ring Laguerre–Gaussian beams with numbers (0, *m*) that are parallel to the optical axis and have the same phase but different power in the initial plane has a topological charge equal to *m*, regardless of the distance between the beams and of the power of each beam. Furthermore, only if the beams have a different phase in the initial plane (in the waist plane), then the superposition *TC* changes. The theoretical consideration is confirmed by the simulation results, which demonstrate how tuning the phase delays between the superposition coefficients allows the *TC* of the superposition to be conserved, or incremented, or decremented. The potential application areas are optical data transmission and optical computing.

## Data Availability

Not applicable.
